# Evaluation of depression and anxiety control in greek patients with major depressive disorder with/without generalized anxiety disorder and cardiovascular disease–pronoi study

**DOI:** 10.1192/j.eurpsy.2021.266

**Published:** 2021-08-13

**Authors:** P. Papanastasiou, P. Gourzis, I. Nimatoudis, A. Ginis

**Affiliations:** 1 Medical Department, ELPEN Pharmaceutical Co. Inc., Attica, Greece; 2 Department Of Psychiatry, School of Medicine, University of Patras, Patras, Greece; 3 Third Department Of Psychiatry, Aristotle University of Thessaloniki, Thessaloniki, Greece

**Keywords:** Depression, Anxiety, comorbidities, cardiovascular

## Abstract

**Introduction:**

Patients with depression are likely to eventually develop Cardiovascular disease(CVD) and have a higher mortality rate than general population. In addition, anxiety disorders, especially Generalized Anxiety Disorder (GAD), may be associated with mortality and other adverse cardiac outcomes.

**Objectives:**

Evaluation of depression and anxiety control in Greek patients with Major Depressive Disorder (MDD) with/without GAD and CVD, under 6 months of treatment with citalopram, and/or quetiapine, and/or pregabalin.

**Methods:**

565 patients with MDD with/without GAD, enrolled in this observational, study (NCT03317262). The subgroup of 133(24%) patients had CVD. Severity of MDD and GAD symptoms was evaluated using the HAM-D and HAM-A Scores at baseline (V_1_) and after 6 months (V_3_) respectively.

**Results:**

Mean HAM-D score in patients with CVD without GAD, at V_1_ and V_3_ was 23.94±7.51 and 8.14±4.65 respectively (p<0.0001). Similar results were observed in patients without CVD without GAD (HAM-D score 26.67±8.79 at V_1_ and 7.44±4.40 at V_3_). Mean HAM-A score in patients with CVD and GAD at V_1_ and V_3_ was 25.64±6.38 and 8.98±3.93, respectively (p<0.0001). Same magnitude reduction in HAM-A score was observed in patients without CVD and GAD, 26.27±8.16 at V_1_ and 9.28±6.48 at V_3_ (p<0.0001). Patients’ depression symptoms with/without CVD and GAD showed also a significant reduction between V_1_ and V_3_.

**Conclusions:**

MDD patients with CVD without GAD, had a marginally lower baseline HAM-D score versus patients with GAD. After 6 months of treatment with citalopram, and/or quetiapine, and/or pregabalin the improvement of depressive and anxiety symptoms was almost equal between MDD patients with/without GAD regardless of the presence of coexisting CVD.

**Disclosure:**

Employee of ELPEN Pharmaceutical Co. Inc.

